# Non-canonical Glucocorticoid Receptor Transactivation of *gilz* by Alcohol Suppresses Cell Inflammatory Response

**DOI:** 10.3389/fimmu.2017.00661

**Published:** 2017-06-07

**Authors:** Hang Pong Ng, Scott Jennings, Jack Wang, Patricia E. Molina, Steve Nelson, Guoshun Wang

**Affiliations:** ^1^Alcohol and Drug Abuse Center, Department of Microbiology, Immunology and Parasitology, Louisiana State University Health Sciences Center, New Orleans, LA, United States; ^2^Department of Physiology, Louisiana State University Health Sciences Center, New Orleans, LA, United States; ^3^Department of Medicine, Louisiana State University Health Sciences Center, New Orleans, LA, United States

**Keywords:** glucocorticoid receptor, glucocorticoid-induced leucine zipper, glucocorticoid, alcohol, LPS, inflammatory response

## Abstract

Acute alcohol exposure suppresses cell inflammatory response. The underlying mechanism has not been fully defined. Here we report that alcohol was able to activate glucocorticoid receptor (GR) signaling in the absence of glucocorticoids (GCs) and upregulated glucocorticoid-induced leucine zipper (*gilz*), a prominent GC-responsive gene. Such a non-canonical activation of GR was not blocked by mifepristone, a potent GC competitor. The proximal promoter of *gilz*, encompassing five GC-responsive elements (GREs), was incorporated and tested in a luciferase reporter system. Deletion and/or mutation of the GREs abrogated the promoter responsiveness to alcohol. Thus, the GR–GRE interaction transduced the alcohol action on *gilz*. Alcohol induced GR nuclear translocation, which was enhanced by the alcohol dehydrogenase inhibitor fomepizole, suggesting that it was alcohol, not its metabolites, that engendered the effect. Gel mobility shift assay showed that unliganded GR was able to bind GREs and such interaction withstood clinically relevant levels of alcohol. GR knockout *via* CRISPR/Cas9 gene targeting or GILZ depletion *via* small RNA interference diminished alcohol suppression of cell inflammatory response to LPS. Thus, a previously unrecognized, non-canonical GR activation of *gilz* is involved in alcohol modulation of cell immune response.

## Introduction

Ethanol, commonly referred to alcohol in everyday life, is the intoxicating ingredient found in beer, wine, and liquor. Alcohol is one of the most consumed substances and affects human health positively or negatively depending on how it is used. Moderate alcohol intake reduces the risk of many adverse health conditions including coronary artery disease, diabetes, hypertension, congestive heart failure, stroke, arthritis, and dementia (1–3). However, alcohol abuse is linked to organ and tissue damage (3–5), which often leads to life-threatening medical complications (6, 7). Alcohol has long been recognized as a potent immunosuppressive agent that predisposes individuals to infections by bacteria, fungi, and viruses (3, 8–11) and leads to specific defects in innate and adaptive immunity (6, 12).

The hypothalamic–pituitary–adrenal (HPA) axis is a major regulatory system of host immunity, which is accomplished by adrenal production of glucocorticoids (GCs) (13). GCs act through intracellular interaction with glucocorticoid receptor (GR), a ligand-dependent transcriptional factor that belongs to the superfamily of steroid/thyroid/retinoic acid receptor proteins (14–16). GR signaling is crucial to modulating host immunity. Clinically, steroid therapy is the most prescribed medication for treating chronic inflammatory conditions and autoimmune diseases (13, 17, 18) and animals with conditional GR-knockout succumb to LPS-induced septic shock (19). GR consists of an amino-terminal transactivation domain, a central DNA-binding domain, and a carboxy-terminus containing the hormone-binding domain as well as sequences related to nuclear translocation, receptor dimerization, and interaction with other cellular proteins (20). In the absence of ligands, GR is sequestered in the cytoplasm by various accessory proteins, including hsp90, hsp70, hsp56, p23, and immunophilins (21). Upon hormonal binding, the receptor undergoes a conformational change and translocates into the cell nucleus where it binds to GC-responsive elements (GREs) in the promoters of GR-targeting genes (22) or tethers with other protein factors (23). Among the GR-targeting genes, Glucocorticoid-induced leucine zipper (GILZ) is a member of the transforming growth factor-beta (TGF-β)-stimulated clone-22 (TSC-22) family of transcription factors (24). Direct evidence for the role of GILZ in host immune suppression comes from the naturally evolved inbred SPRET/Ei mice, which are resistant to LPS-induced endotoxemia as a result of an intrinsically increased production of GILZ due to a genetic variation (25). Macrophages with GILZ-knockout lose their LPS tolerance (26). GILZ is reported to transduce the anti-inflammatory action of GCs in epithelial cells (27) and redirects the maturation of human dendritic cells to prevent antigen-specific T lymphocyte response (28). Transgenic mice overexpressing GILZ downregulate the Th-1 and upregulate the Th-2 responses (29–31). All these data implicate GILZ as a key player in host anti-inflammation and immunosuppression (31–33).

Alcohol was reported to elevate plasma GC concentration at the physiological level (34, 35), which activates GR signaling through the conventional GC–GR interaction. However, whether alcohol has direct impact on GR signaling at the cellular level is unknown. An early study by Mandrekar and colleagues demonstrated that acute alcohol increases nuclear translocation of non-ligand-bound GR (36). However, whether this unliganded GR has any biological significance was not defined in that study. Our genome-wide analysis of alcohol influence of gene expression demonstrated that acute alcohol upregulates a cluster of GC-responsive genes in a dose-dependent manner (37), including GILZ (TSC22D3) (31), ALOX15B (38), SYNPO2 (39), and PTEN (40), implying that the alcohol-induced non-ligand-bound GR is biologically functional. The current study was designed to delineate the mechanism underlying how alcohol influences GR signaling and activates GC-responsive genes, such as GILZ, in the absence of GC ligands.

## Materials and Methods

### Chemicals and Reagents

Common reagents were from Sigma-Aldrich (St. Louis, MO, USA). Alcohol or ethanol (200 proof, absolute ACS/USP Grade) was purchased from Pharmco Products Inc. (Shelbyville, KY, USA). Radioactive γ-^32^P-ATP was obtained from Perkin Elmer (Billerica, MA, USA). All oligonucleotides were ordered from Integrated DNA Technologies (Coralville, IA, USA) and their sequences are displayed in Table 1.

**Table 1:** Oligonucleotides used in experiments.

Name	Oligonucleotide sequence
GILZ-F	5′-CATGGAGGTGGCGGTCTATC-3′
GILZ-R	5′-CACCTCCTCTCTCACAGCGT-3′
Long-GILZp-F	5′-CGGGGTACGTGCAGAGGGCAAATTAATA-3′
Short-GILZp-F	5′-GGGAATTCTGATACCGGCATAACTGCCCTG-3′
GILZp-R	5′-CCCAAGCTTCGCAGTCCAACCCAGACTC-3′
Mut-GRE1-F	5′-CAAACTAGCGCCAGGTCTTACTGAATGCCC-3′
Mut-GRE1-R	5′-GGGCATTCAGTAAGACCTGGCGCTAGTTTG-3′
Mut-GRE2-F	5′-GGTGGAACCCAAGAGCACCCTTTGGTCCTG-3′
Mut-GRE2-R	5′-CAGGACCAAAGGGTGCTCTTGGGTTCCACC-3′
Wt-GRE1(+)	5′-CAAACTAGCGCTGTTCTTTACTGAATGCCC-3′
Wt-GRE1(−)	5′-GGGCATTCAGTAAAGAACAGCGCTAGTTTG-3′
Mut-GRE1(+)	5′-CAAACTAGCGCCAGGTCTTTACTGAATGCCC-3′
Mut-GRE1(−)	5′-GGGCATTCAGTAAAGACCTGGCGCTAGTTTG-3′
Wt-GRE2(+)	5′-GGTGGAACCCAATGTTCTCCTTTGGTCCTG-3′
Wt-GRE2(−)	5′-CAGGACCAAAGGAGAACATTGGGTTCCACC-3′
Mut-GRE2(+)	5′-GGTGGAACCCAAGAGCACCCTTTGGTCCTG-3′
Mut-GRE2(−)	5′-CAGGACCAAAGGGTGCTCTTGGGTTCCACC-3′
GR-E3 (+)	5′-GAGTTCACTGTGAGCATTC-3′
GR-E3 (−)	5′-GAGGGTAAGGATCAGGTCTTATTG-3′

### Cell Culture and Alcohol Exposure

A549 and Mono Mac 6 (MM6) cells were, respectively, cultured in Dulbecco’s Modified Eagle Medium (Gibco) and advanced RPMI-1640 (Invitrogen, Carlsbad, CA, USA) supplemented with 10% fetal bovine serum (FBS) (HyClone, Logan, UT, USA), 2 mM GlutaMax (Gibco), 100 U/ml penicillin, 100 µg/ml streptomycin, and 0.25 µg/ml amphotericin B. Forty-eight hours before and during experiments, fresh media with 10% charcoal-stripped FBS (Atlanta Biological) were used to avoid GC interference in experiments. For alcohol exposure, various doses (0, 25, or 50 mM) of alcohol were added to the culture media, respectively, and the cells were cultured in the incubators presaturated with the corresponding levels of alcohol.

### MM6 Cell Treatments and RT-qPCR for Gilz Expression

MM6 cells (1.5 × 10^6^) were exposed to alcohol (0, 25, or 50 mM) or 1 µM Dexamethasone (Dex) (Sigma-Aldrich) for 24 h in the presence or absence of 5 µM mifepristone (Sigma-Aldrich). The cells were harvested and washed with 1× PBS twice. Total RNAs were extracted using the Qiagen RNeasy Kit. The cDNAs were synthesized using the Quantitect Reverse Transcriptase Kit (Qiagen). Human GILZ primers (GILZ-F and GILZ-R) and glyceraldehyde 3-phosphate dehydrogenase (GAPDH) primers (GAPDH-F and GAPDH-R) were used at a final concentration of 500 nM. The final reaction for each sample was brought to a total volume of 20 µl with RT SYBR green qPCR mastermixes (Qiagen). All reactions were carried out in duplicate on a CFX96 system (Bio-Rad Laboratories, Hercules, CA, USA) for quantitative real-time PCR (qPCR) detection. The qPCR data were analyzed by the comparative Ct (ΔΔCT) method. The expression of GILZ of each treated group was compared to that of GAPDH and normalized to the non-treatment group.

### Luciferase Plasmid Construction

The *gilz* promoter was PCR-amplified from the genomic DNA of primary human airway epithelial cells (Lonza Walkersville, Inc., Walkersville, MA, USA) using the primers (Long-GILZp-F and GILZp-R). The amplicon was cloned into pCR™-Blunt II-TOPO^®^ plasmid (Invitrogen), and the primary sequence was verified by DNA sequencing. Then, the obtained 1.94-Kb promoter was excised with *Kpn*I/*Hin*dIII and subcloned into the promoter-less pGL.4.16[*luc2CP/*Hygro] plasmid (Promega, Madison, WI, USA), resulting in pGL.4.16-wtGILZ-2p-[*luc2CP/*Hygro] in which the GILZ promoter drives the expression of the rapid-response *firefly* luciferase (Fluc) gene. The cloned promoter contains five GREs. To remove GRE3-5, the region (−1,560 to −1,884) was deleted using the primers (Short-GILZp-F and GILZp-R). To achieve site-specific mutagenesis of the GRE1 and GRE2 core sequences, QuikChange II Site-Directed Mutagenesis Kit (Agilent Technologies) was used, as instructed by the manufacturer. Synthetic oligonucleotide primers (Mut-GRE1-F, Mut-GRE1-R, Mut-GRE2-F, and Mut-GRE2-R) were used to introduce mutations. All the plasmids with mutagenesis were verified by DNA sequencing.

### Dual-Luciferase Reporter Assay

Transfection was performed with Lipofectamine 2000 Transfection Reagent (Invitrogen, Carlsbad, CA, USA) according to the manufacturer’s instruction. Each of the constructed plasmids was cotransfected into A549 cells with the reference plasmid pGL4.73[hRluc/SV40] (Promega) that expresses *Renilla* luciferase. Forty-eight hours later, the transfected cells were exposed to 0 or 50 mM of alcohol or 1 µM Dex for 24 h. Activities of *Firefly* luciferase and *Renilla* luciferase were measured using the dual luciferase assay system (Promega). Potency of the wild-type or mutant *gilz* promoter to drive the reporter luciferase expression was determined by ratio of the activity of *Firefly* luciferase over that of *Renilla* luciferase.

### Gel Mobility Shift Assay (GMSA)

Double-stranded oligonucleotides for GRE1 and its flanking region and for GRE2 and its flanking region were prepared and end-labeled with ^32^P using T4 polynucleotide kinase. GMSA was performed by incubation of recombinant human GR protein (40 ng or 0.5 pmol) (Sigma-Aldrich), ^32^P-labeled oligonucleotides (0.5 ng or 0.05 pmol) and 1 µg poly-deoxyinosinic-deoxycytidylic acid (Sigma-Aldrich), in a final volume of 25 µl for each reaction. The GR-GRE complex was resolved by non-denature polyacrylamide gel electrophoresis and detected by X-ray film exposure.

### GILZ Knockdown in MM6 Cells

Lentiviral vectors expressing either siGILZ or siCNTL transgene were constructed as previously described (37) and were produced *via* triple-plasmid cotransfection of HEK 293T cells (41). Transduction of MM6 cells was carried out at MOI of 5 in the presence of Polybrene (Sigma-Aldrich, 8 µg/ml). Because the lentiviral vectors had an EGFP marker gene in an independent cassette, each set of vector-transduced cells was sorted through multiple rounds to a pure population.

### GR Knockout *via* CRISPR/Cas9 Gene Targeting

The *S. pyogenes* Cas9 plasmid pSpCas9(BB)-2A-GFP (PX458) (Addgene plasmid # 48138) was a gift from Dr. Feng Zhang at the Massachusetts Institute of Technology. A target-specific sgRNA sequence for Exon 3 of human GR gene was identified using the CRISPR MultiTargeter^1^ and the Zhang lab target finder^2^ online tools. The target sgRNA sequence was cloned into the Cas9 vectors according to the Zhang lab protocol (42). In brief, two single-stranded DNA oligos were designed for the sgRNA target (oligo 1: 5′-CACCGCATTAT-GGAGTCTTAACTTG-3′; oligo 2: 5′-AAACCAAGTTAAGACTCCATAATGC-3′). The pair of oligos was phosphorylated and annealed with T4 Polynucleotide Kinase (NEB). The Cas9 plasmids were digested with *Bbs*I (NEB) and then ligated with the annealed oligos using NEB Quick Ligase. The final constructed plasmids were sequenced for verification. MM6 cells (1.4 × 10^6^) in 300 µl of RPMI 1640 media were combined with 20 µg of Cas9-GFP-GR plasmid, incubated on ice for 20 min and electroporated *via* exponential decay pulse (200 V, 975 μF) using a Bio-Rad Gene Pulser Xcell system. Following electroporation, cells were incubated on ice again for 20 min and cultured in complete RPMI 1640 overnight. Then, the cells were sorted for GFP-positive population using FACS. The sorted cells were plated on 96-well culture plates at single-cell density. After 7–14 days of growth, cell clones were screened by PCR using GR-E3(+) and GR-E3(−) primers and DNA sequencing to confirm GR gene knockout.

### Western Blot Assay

For nuclear GR measurement, MM6 cells were exposed to alcohol (0 or 50 mM) or Dex (1 µM) for 24 h in the presence or absence of fomepizole (4 µM). The cell nuclei were isolated using Thermo Scientific™ NE-PER™ Nuclear and Cytoplasmic Extraction Reagents in accordance with the manufacturer’s instruction. The samples were then subjected to Western blot. Rabbit polyclonal antibodies against human GR (Santa Crus Technology, Santa Cruz, CA, USA) and Histone H3 (Cell Signaling Technology, Danvers, MA, USA) were used. Intensities of the GR and Histone H3 bands were estimated by densitometry using NIH Image-J system.

For GILZ measurement, parental MM6, siCNTL-MM6, and siGILZ-MM6 cells were, respectively, treated with 0 or 1 µM Dex for 24 h. Cells were collected, harvested, and lysed with 1× RIPA buffer (Thermo Fisher Scientific, Waltham, MA, USA) supplemented with 1× protease inhibitor cocktail (Roche, Indianapolis, IN, USA). The cell lysates were collected and subjected to BCA protein assay (Thermo Fisher Scientific, Waltham, MA, USA) for protein concentration. Proteins (50 µg) from each sample were loaded onto 10–20% Tris–Tricine precast gel (Bio-Rad, Hercules, CA, USA) in the Mini-Protein system (Bio-Rad) for electrophoresis. Rabbit polyclonal anti-human GILZ antibody at 1:200 final dilution (FL-134, Santa Cruz Biotechnology) and rabbit anti-β-Actin antibody at 1:5,000 final dilution (Cell Signaling Technology, Danvers, MA, USA) were used to probe GILZ and actin.

### LPS Stimulation and TNF-α Measurement

Different genotypes of MM6 cells, as indicated in each experiment, were exposed to alcohol (0 or 50 mM) 1 h before stimulation with 100 or 1,000 ng/ml LPS from *Escherichia coli* 0111:B4 (Sigma-Aldrich). The supernatants were collected at different time points. TNF-α was measured by ELISA (R&D Systems) following the manufacturer’s recommendations.

### Statistical Analysis

Statistical analysis was performed with Prism 7.0 (GraphPad Software, La Jolla, CA, USA). A 2-tailed, unpaired Student’s *t*-test was used for statistical comparisons. All the data are expressed as mean ± SD. Difference with *p*-value smaller than 0.05 is considered statistically significant.

## Results

### Alcohol Activates GR Signaling *via* a Non-Canonical Mechanism

**Figure 1:**
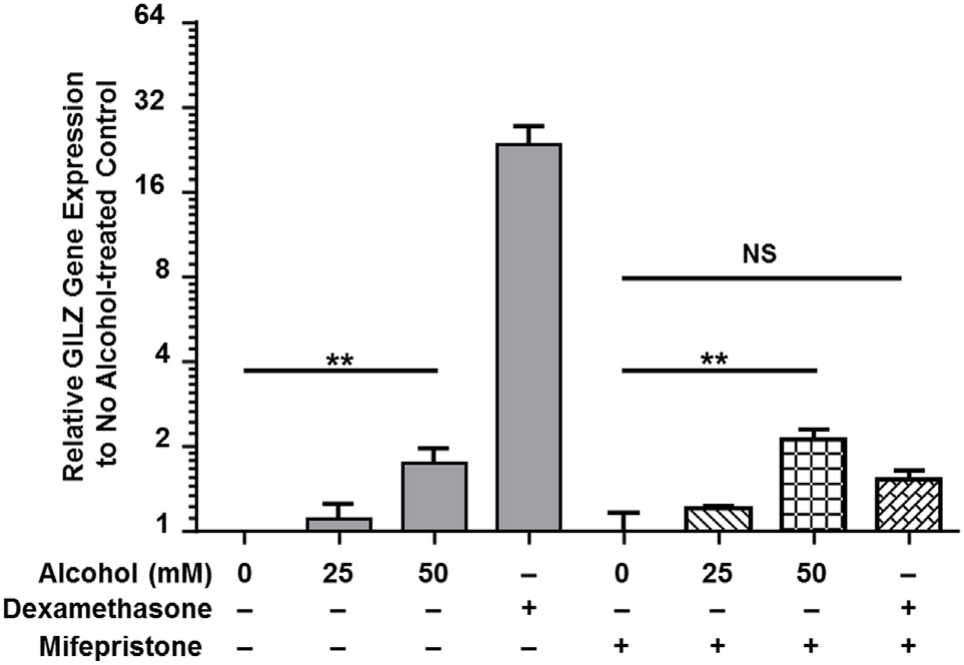
Mifepristone blocks dexamethasone- but not alcohol-activation of gilz. MM6 cells were exposed to alcohol (0, 25, or 50 mM) or dexamethasone (Dex, 1 µM) for 24 h in the presence or absence of mifepristone (5 µM). GILZ expression from each treatment was measured by quantitative real-time PCR. Changes in GILZ expression were expressed by fold increases as compared to the base level of GILZ in the no treatment control. The data represent at least three independent experiments. The shown *P*-values by Student’s *t*-test indicate statistically significant differences. NS denotes non-significant difference.

Classical activation of GR signaling is initiated by GC binding to GR that resides in the cytoplasm in complex with various chaperone proteins. Then, GR undergoes a conformational change, dissociates from the complex, and translocates to the nucleus to modulate gene transcription (43). However, our previously published data demonstrated that alcohol upregulates *gilz*, a prominent GC-responsive gene, in the absence of GC (37), suggesting an alternative GR-activation mechanism under alcohol exposure. To delineate the mechanism, we exposed MM6 cells to various concentrations of alcohol (0, 25, or 50 mM) or dexamethasone (Dex, 1 µM) for 24 h in the presence or absence of the GC competitor mifepristone (5 µM). Transcription of *gilz* was assessed by quantitative real-time-PCR. As shown in Figure 1, alcohol (50 mM) significantly upregulated *gilz* by ~2-fold. Dex increased *gilz* expression by ~27-fold. Importantly, mifepristone completely blocked Dex-activation of *gilz*, but had no effect on alcohol-activation of *gilz*. Because Dex is a synthetic GC that activates GR signaling as a GR agonist, while mifepristone a potent competitive GR antagonist, the data indicate that the mechanism for alcohol activation of GR is different from that for GC activation of GR.

### Alcohol Activates Gilz Promoter through GR–GRE Interaction

To define the *cis*-acting elements that are responsible for alcohol activation of *gilz*, we cloned the 1.94-kb promoter region of *gilz*. Primary sequence analysis by the CISTER algorithm method (44) revealed multiple clusters of *cis*-elements, including GRE, cAMP response element (CRE), Forkhead responsive element (FHRE-1), serum response element (SRE), signaling transducer and activator of transcription 6 (STAT-6), GATA Box (GATA), and TATA box (TATA) (Figure S1 in Supplementary Material). Noticeably, there are five GREs, three of which (GRE3, 4, and 5) are relatively distal, and the other two (GRE1 and 2) proximal to the transcriptional initiation site defined by a previous study (45).

**Figure 2:**
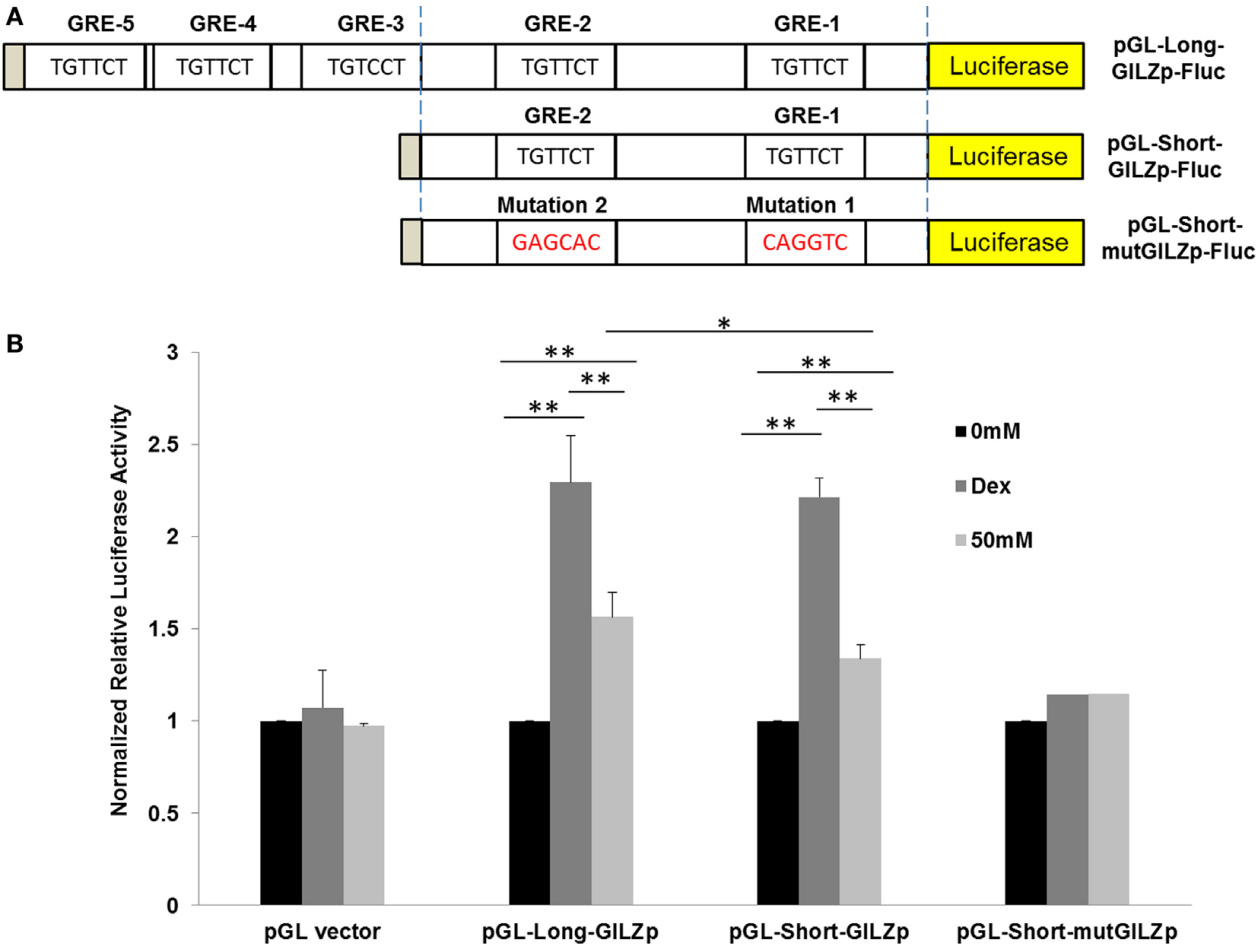
Alcohol activates GILZ promoter *via* GRE cis-elements. **(A)** The *gilz* promoter was cloned into the promoter-less pGL.4.16[luc2CP/Hygro] construct, generating the pGL-Long-GILZp-Fluc plasmid. GRE3-5 sites were removed by deletion of the region (−1,560 to −1,884), resulting in the pGL-Short-GILZp-Fluc plasmid. The GRE1 and GRE2 sites were mutated, producing the pGL-Short-mutGILZp-Luc plasmid. **(B)** A549 cells were, respectively, transfected with one of the three resulting plasmids in combination with the reference plasmid pGL4.75-hRluc that constitutively expressed the humanized *Renilla* luciferase. Forty-eight hours after transfection, the cells were exposed to 0 or 50 mM alcohol or 1 µM of Dex for 24 h. Potency of the wild-type or each mutated *gilz* promoter to drive the reporter gene expression was determined by the activity of the *Firefly* luciferase that had been normalized to that of the *Renilla* luciferase in each sample. Asterisks denote significant differences between the comparing groups by Student’s *t*-test (*p < 0.05, **p < 0.01; *n* = 3).

To compare Dex-activation of *gilz* with alcohol activation of *gilz*, we subcloned the 1.94-kb *gilz* promoter into the promoter-less pGL.4.16[*luc2CP/*Hygro] construct, generating the pGL-Long-GILZp-Fluc plasmid (Figure 2A), in which the cloned promoter was designed to regulate the expression of the rapid-response *firefly* luciferase (Fluc) gene. Because GRE3, 4, and 5 were located closely, we decided to remove them altogether by deletion of the region (−1,560 to −1,884), resulting in a shorter *gilz* promoter and the pGL-Short-GILZp-Fluc plasmid. On the basis of this short promoter construct, we further site-mutated the proximal GRE1 and GRE2 core sequences, producing the pGL-Short-mutGILZp-Luc plasmid (Figure 2A) that had lost all five GREs. A549 cells were, respectively, transfected with one of the three generated plasmids in combination with the reference plasmid pGL4.75-hRluc that constitutively expressed the humanized *Renilla* luciferase. Forty-eight hours after transfection, the cells were exposed to 0 or 50 mM alcohol or 1 µM of Dex for 24 h. Potency of the wild-type or the mutated *gilz* promoter to drive the reporter gene expression was determined by the activity of the *Firefly* luciferase that had been normalized to that of the *Renilla* luciferase in each sample. The data (Figure 2B) demonstrate that the promoter-less pGL.4.16-[*luc2CP/*Hygro] vector (pGL-vector) did not respond to either Dex or alcohol stimulation. However, the long wild-type *gilz* promoter with all five GREs (pGL-Long-GILZp) faithfully responded to Dex and alcohol, displaying ~2.3- and ~1.6-fold higher *firefly* luciferase activity than the non-treatment control. Deletion of GRE3, 4, and 5 (pGL-Short-GILZp) significantly reduced the *gilz* promoter responsiveness to alcohol but not Dex. Further mutations of GRE1 and GRE2 (pGL-Short-mut-GILZp) totally abolished the responsiveness of the promoter to both Dex and alcohol. These data suggest that alcohol bypasses GC to activate *gilz* promoter through the GRE *cis*-elements, and that all five GREs appear to be needed for a full action of alcohol. In contrast, the GRE1 and GRE2 are sufficient to convey the full effect of Dex.

### Alcohol Instead of Alcohol Metabolites Enhances GR Nuclear Translocation

**Figure 3:**
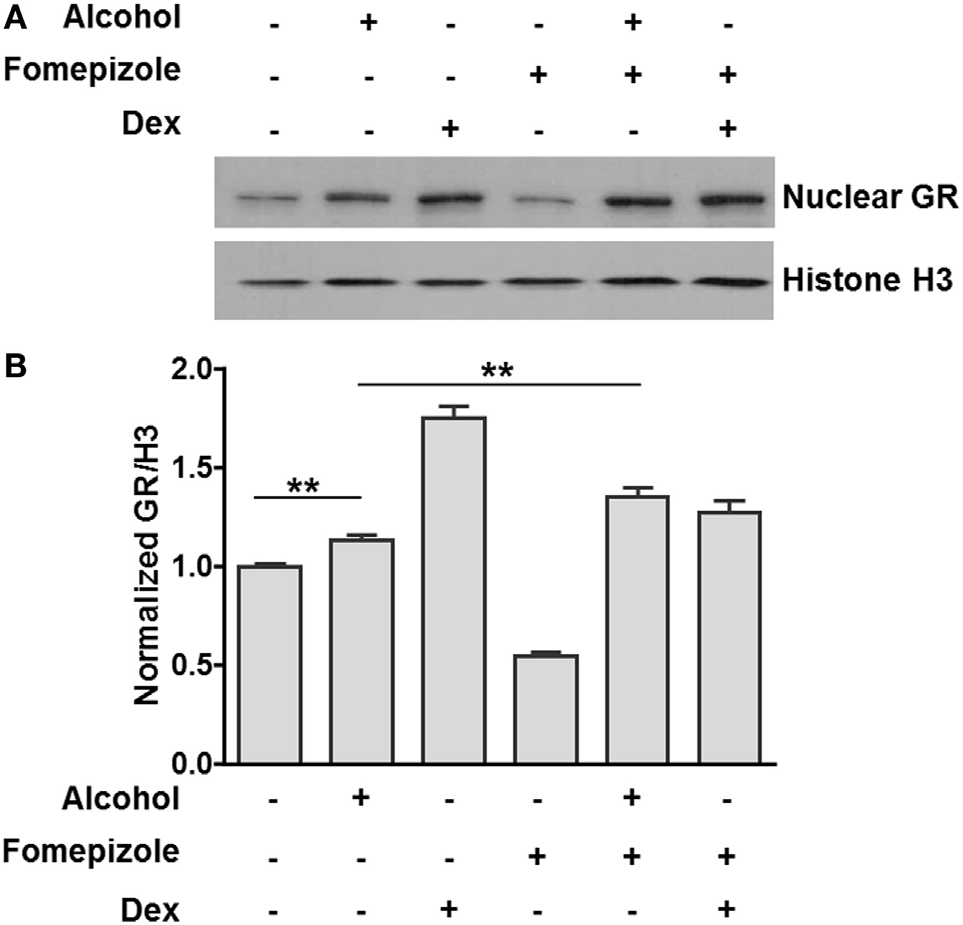
Alcohol dehydrogenase (ADH) inhibitor fomepizole enhances GR nuclear translocation. **(A)** Western blots of nuclear GR and Histone H3. MM6 cells were exposed to alcohol (0 or 50 mM) or dexamethasone (Dex, 1 µM) for 24 h in the presence or absence of the ADH inhibitor fomepizole (4 µM). Cell nuclei from each treatment were isolated and subjected to Western blot analysis. Histone H3 was also assessed for loading normalization. **(B)** Statistical data from three independent experiments. The intensities of the GR and Histone H3 bands were quantified by densitometry. Ratio of nuclear GR over the corresponding Histone H3 for each treatment was obtained and graphed. Significant difference was judged by Student’s *t*-test (*p* < 0.01; *n* = 3).

Previous publications from our laboratory and others have reported that alcohol induces nuclear translocation of non-ligand-bound GR (35, 36). However, it is unknown whether this effect is caused by alcohol itself or alcohol metabolites. We exposed MM6 cells to alcohol (0 or 50 mM) for 24 h in the presence or absence of the alcohol dehydrogenase (ADH) inhibitor fomepizole (4 µM). The levels of nuclear GR and Histone H3 for each condition were assessed by Western blot assay and densitometry quantification (Figure 3A). The ratio of nuclear GR over Histone H3 for each condition was calculated. Data from three separate experiments (Figure 3B) show that alcohol exposure increased nuclear GR as compared to that of the non-alcohol control. Strikingly, fomepizole enhanced GR nuclear translocation. Thus, alcohol-induced GR nuclear translocation is likely the effect of alcohol molecules instead of alcohol-derivatives.

### Unliganded GR Is Able to Bind GREs in the Presence of Clinically Relevant Levels of Alcohol

**Figure 4:**
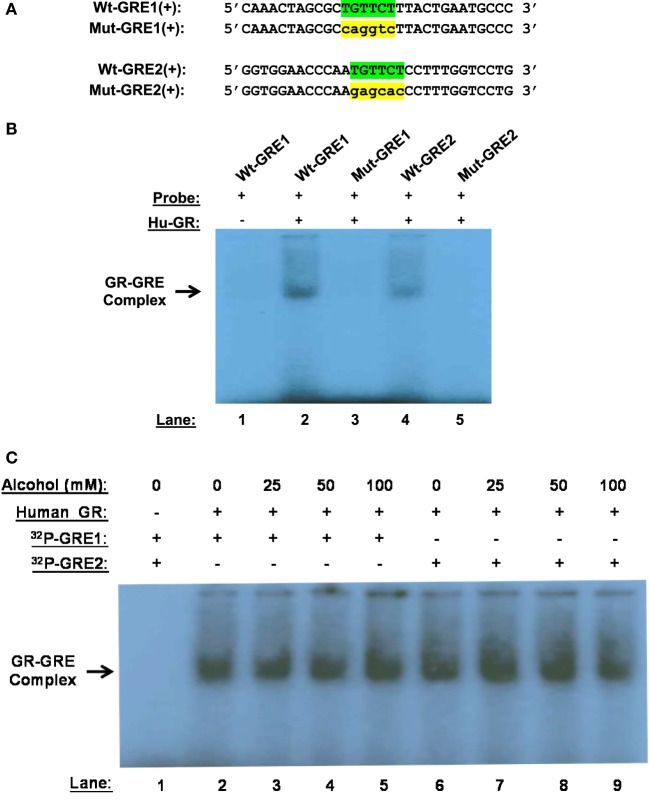
Gel mobility shift assay (GMSA). **(A)** Probe sequences. Sequences of the positive strand of Wt-GRE1, Mut-GRE1, Wt-GRE2, and Mut-GRE2 are displayed. **(B)** GR–GRE binding. Human recombinant GR protein (40 ng, Hu-GR) was incubated with each of the following double-stranded probes: wild-type GRE1 (Wt-GRE1), mutant GRE1 (Mut-GRE1), wild-type GRE2 (Wt-GRE2), or mutant GRE2 (mut-GRE2). After resolving in a 6% polyacrylamide non-denaturing gel, the GR–GRE complex was detected by X-ray film exposure. **(C)** Alcohol effect on GR-GRE binding. The GR protein (40 ng, Hu-GR) was incubated with the radio-labeled wild-type GRE1 or GRE2 probe in the presence of various concentrations of alcohol (0, 25, 50, or 100 mM). GR–GRE binding was similarly examined by GMSA.

Alcohol induces GR–GRE interaction in the absence of GC, predicting that non-ligand-bound GR can bind GREs. To prove the prediction, we synthesized and radioactively labeled the double-stranded oligonucleotides covering the core and flanking sequences of GRE1 and GRE2 (Figure 4A). Similarly, the corresponding core-sequence-mutated GRE1 and GRE2 oligonucleotides were also prepared. GMSA was undertaken by incubation of human GR protein with the radioactive oligonucleotides. Non-denaturing polyacrylamide gel electrophoresis revealed that human GR protein without GC coupling formed a GR–GRE complex with the wild-type GRE1 and GRE2 oligonucleotides, but not the mutant counterparts (Figure 4B). This result proves that non-ligand-bound GR is able to interact with GRE in the absence of ligands.

We further tested if the interaction between the unliganded-GR and GRE could withstand alcohol at clinically encountered levels. Human GR protein was incubated with the wild-type GRE1 or GRE2 probe in the presence of alcohol (0, 25, 50, or 100 mM). GMSA revealed that the non-canonical GR-GRE interaction was strong enough to withstand all the tested levels of alcohol (Figure 4C).

### GR Knockout or GILZ Knockdown Diminishes Alcohol Suppression of Cell Inflammatory Response

**Figure 5:**
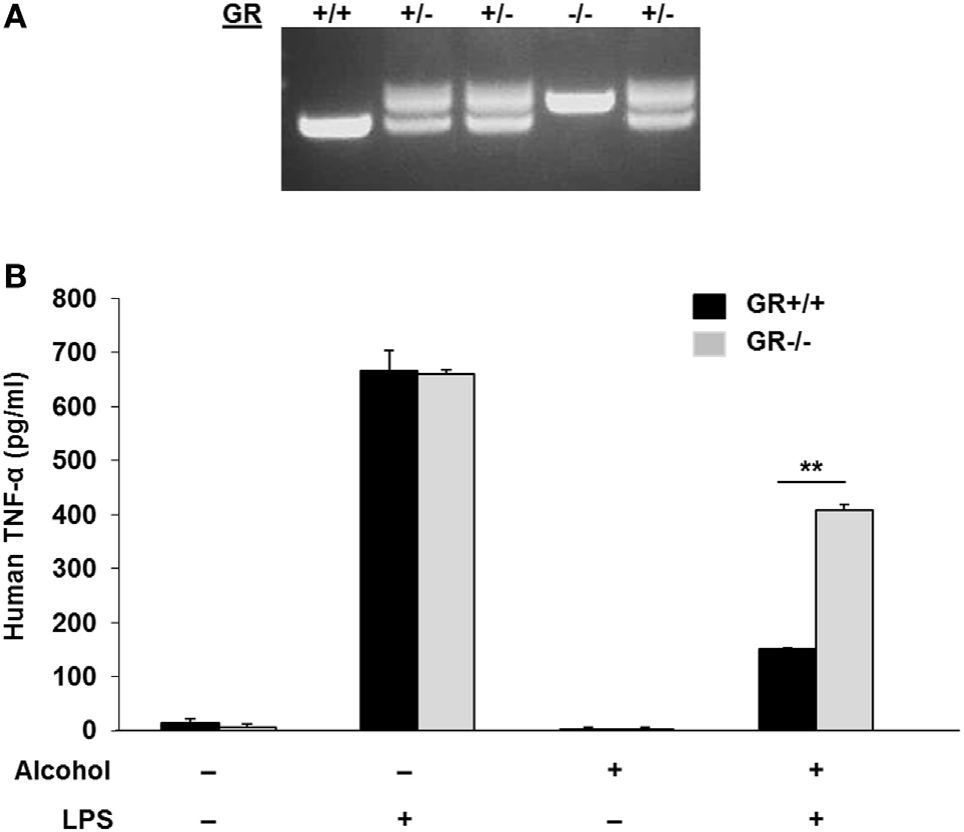
Glucocorticoid receptor (GR) knockout in MM6 cells diminishes alcohol suppression of cell inflammatory response to LPS. **(A)** GR gene knockout in MM6 cells. CRISPR/Cas9 gene targeting was used to knockout the GR gene. PCR amplification of the targeted GR Exon 3 and its flanking sequences shows multiple cell clones with wild-type (GR+/+), heterozygote (GR±) and homozygote (GR−/−). The GR−/− cell clone has a 94-bp insertion mutation (see Figure S2 in Supplementary Material), which resulted in a truncated GR (see Figure S3 in Supplementary Material). **(B)** GR+/+ and GR−/− MM6 cells (1 × 10^6^) were exposed to 0 or 50 mM alcohol and challenged with or without LPS (1 µg/ml) for 24 h. The culture media were collected for TNF-α measurement by ELISA. Asterisks denote statistically significant difference (*p* < 0.01; *n* = 4) by Student’s *t*-test.

To test if *gilz* gene activation by alcohol through the GR-GRE interaction has any functional alteration, we created a GR−/− MM6 cell line by CRISPR/Cas9 gene targeting (Figure 5A), in which a 94-bp insertion mutation was made to GR Exon 3 (Figure S2 in Supplementary Material). Such an insertion led to a premature stop of GR translation and a truncated GR (Figure S3 in Supplementary Material). Then, both GR−/− and GR+/+MM6 cells were exposed to either 0 or 50 mM alcohol, followed by LPS challenge. Twenty-four hours after alcohol exposure, the medium from each condition was collected. Because TNF-α is the key hallmark cytokine induced by LPS (46, 47), we elected to use TNF-α as the representative cytokine to indicate the status of the cell inflammatory response. ELISA measurement of TNF-α revealed that without alcohol exposure, both genotypes of MM6 cells produced comparable levels of TNF-α after LPS stimulation (Figure 5B). Alcohol substantially decreased TNF-α production by GR+/+ cells as compared to their non-alcohol control. However, the alcohol suppressive effect on GR−/− cells was significantly attenuated, suggesting GR without ligand coupling is involved in alcohol suppression of cell inflammatory response. To confirm the fidelity of TNF-α in reflecting cell inflammatory response in our experimental system, we were asked to check more inflammatory genes. Toward this end, we checked three more cytokines: IL-1β, IL-6, and IL-10. Like TNF-α, IL-1β, and IL-6 are proinflammatory, while IL-10 is anti-inflammatory. The results demonstrate that alcohol exposure substantially suppressed proinflammatory cytokine (IL-1β and IL-6) production and substantially promoted anti-inflammatory cytokine production by GR+/+ cells. Strikingly, such alcohol effects were diminished in GR−/− cells (Figure S4 in Supplementary Material). These data confirm that TNF-α is a reliable marker to attest inflammatory response in our system and that GR is crucial in alcohol modulation of cell inflammatory response.

**Figure 6:**
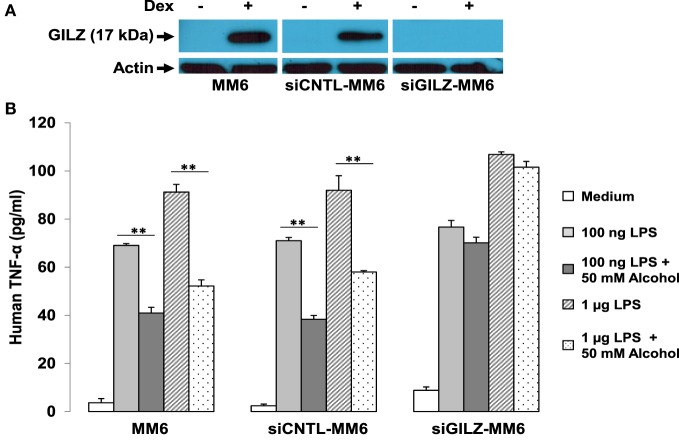
GILZ depletion abrogates alcohol suppression of cell inflammatory response to LPS. MM6 cells were transduced with either the GILZ-knockdown (siGILZ) or the control (siCNTL) lentiviral vector to obtain the stable cell lines with normal GILZ (siCNTL-MM6) or abolished GILZ (siGILZ-MM6) expression. **(A)** Western blot assay to measure GILZ expression. Parental MM6, siCNTL-MM6, and siGILZ-MM6 cells were stimulated with or without Dex (1 µM). The 17-kD GILZ expressions induced by Dex in the parental MM6 and the siCNTL-MM6 cells were examined. Western blot for actin was to show equal loading of all samples. **(B)** GILZ and cell immune response to LPS. Parental MM6, siCNTL-MM6, and siGILZ-MM6 cells were exposed to 0 or 50 mM alcohol, respectively, followed by a challenge with LPS at either 100 ng/ml or 1 µg/ml dose. Twenty-four hours after alcohol exposure, the culture media were collected for measurement of inflammatory cytokine TNF-α. Double asterisks denote significant differences between groups by Student’s *t*-test (*p* < 0.01; *n* = 3).

We further hypothesized that if GR-activation of *gilz* is critical to alcohol suppression of cell inflammatory response, depletion of GILZ should resemble deletion of GR, resulting in abrogation of the alcohol suppressive effect. Taking advantage of the already-made GILZ-knockdown (siGILZ) and control (siCNTL) lentiviral vectors (37), we transduced MM6 cells and created a pair of stable cell lines with expression of a normal level of GILZ (siCNTL-MM6) or a diminished level of GILZ (siGILZ-MM6). To verify GILZ expression in these cells, we performed Western blot assay on the parental MM6, siCNTL-MM6, and siGILZ-MM6 cells stimulated with or without Dex. Because GILZ is a highly GC-responsive gene, Dex application here was to maximally stimulate GILZ expression for the most stringent test of GILZ knockdown. The data (Figure 6A) demonstrate that GILZ (17 kDa) was highly induced by Dex (1 µM) in the parental MM6 and siCNTL-MM6 cells. However, the siGILZ-MM6 cells failed to express any detectable level of GILZ, regardless of Dex induction, indicating that the siRNA effectively eradicates GILZ expression in the GILZ-depleted cells.

To examine whether GILZ depletion in the MM6 cells affects alcohol suppression of the cell inflammatory response, we exposed the parental MM6, siCNTL-MM6, and siGILZ-MM6 cells to 0 or 50 mM alcohol, respectively, 1 h before challenge with LPS at either 100 ng/ml or 1 µg/ml dose. Twenty-four hours after alcohol exposure, the medium from each condition was collected for TNF-α measurement. As shown (Figure 6B), all three types of cells responded to LPS by producing abundant TNF-α in a LPS dose-dependent fashion. Interestingly, GILZ depletion rendered the siGILZ-MM6 cells more inflammatory, producing more TNF-α as compared to the parental MM6 and siCNTL-MM6 cells under an equal level of stimulation. Alcohol exposure at 50 mM substantially suppressed TNF-α production by the parental MM6 and siCNTL-MM6 cells. However, the alcohol-suppressive effect was abolished in the GILZ-depleted cells (siGILZ-MM6), suggesting that GILZ is the major player involved in alcohol suppression of cell inflammatory response to LPS.

### A Proposed Model for Alcohol Activation of GR Signaling

**Figure 7:**
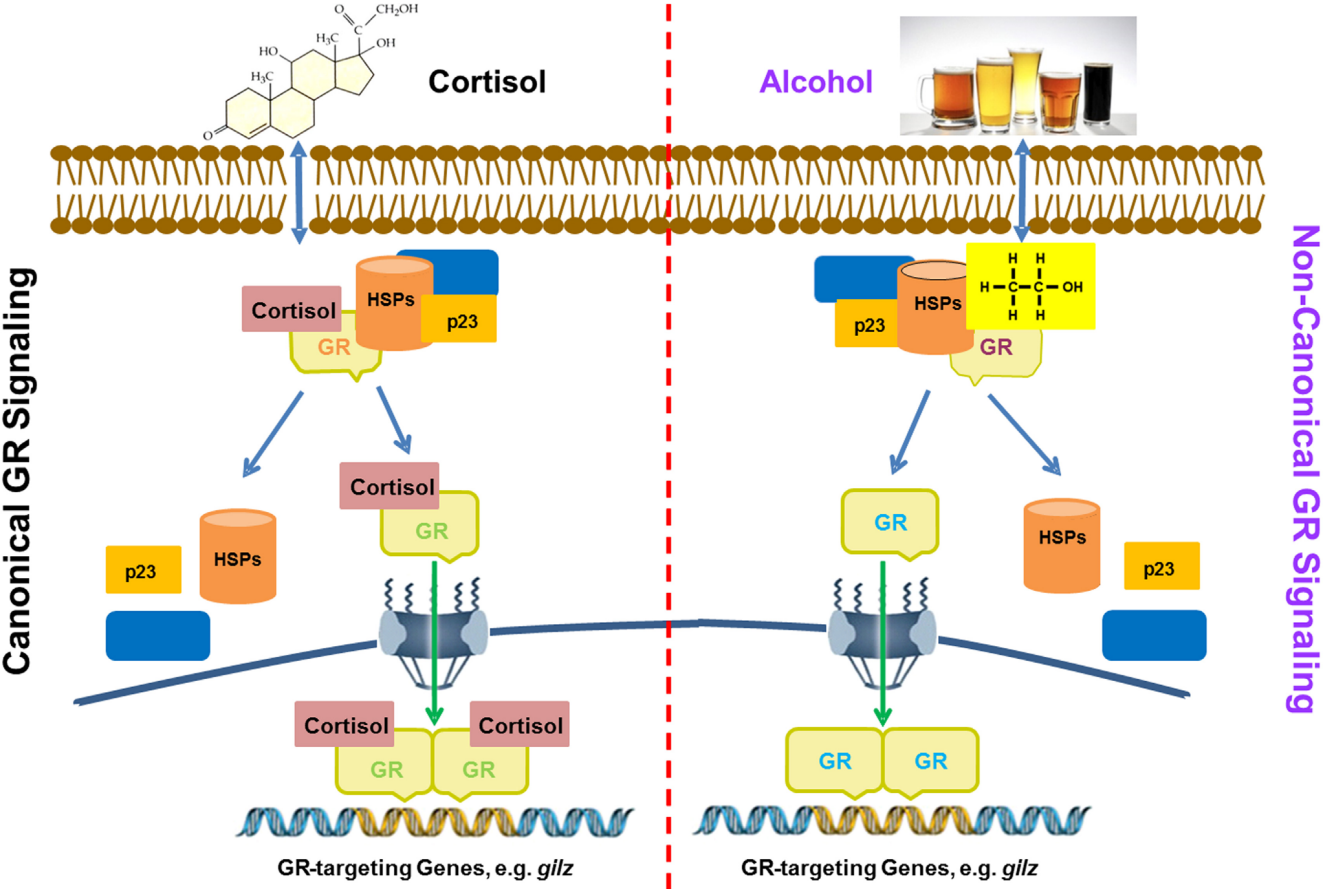
A proposed model for alcohol induction of GIlZ gene expression *via* a non-canonical glucocorticoid receptor (GR) transactivation. Left panel: glucocorticoids (GC) activation of GR signaling. Cortisol as a representative for GCs enters the cytoplasm and binds to GR. Such a binding alters the GR configuration and disassembles the GR complex. Then, the GC-coupled GR is translocated into the nucleus to bind GREs for activation of GR-targeting genes, such as *gilz*. Right panel: Alcohol activation of GR signaling. Alcohol enters the cytoplasm causing disassembly of the GR complex. The freed non-ligand-bound GR migrates to the nucleus and binds GREs for activation of GR-targeting genes, such as *gilz*. Heat shock proteins and p23 represent the chaperone proteins in the cytoplasmic GR complex.

Based on the obtained data, we put forward a working model for alcohol activation of GR signaling, as displayed (Figure 7). Conventional GC-activation of GR signaling starts from GR binding by GC ligands, e.g., cortisol. The GR–GC interaction unleashes GR from the confines of chaperone proteins, such as various heat-shock proteins (HSPs), p23, and others. Then, the GC-coupled GR migrates to the cell nucleus to modulate the expressions of GC-responsive genes. In contrast, alcohol interferes with the stability of the GR complex, from which the unliganded GR is released. Such a non-ligand-bound GR is able to interact with GREs in the promoters of GR-targeting genes including *gilz*. As compared to the conventional GR activation, the alcohol-triggered GR signaling does not involve GCs. This represents a novel non-canonical mechanism for GR activation by alcohol, which may play a pivotal role in alcohol anti-inflammation and immunosuppression.

## Discussion

Alcohol is chemically defined as any organic compound in which a hydroxyl group (–OH) is bound to a carbon atom of an alkyl or substituted alkyl group. Alcohol or ethyl alcohol or ethanol, the active component in all wines and alcoholic beverages, is a simple short-chain alcohol. Even though alcohol is the oldest drug used in medicine and a substance widely consumed for leisure and recreation, mechanistic understanding of its many effects on the human body remains remarkably limited. Alcohol has long been known to be anti-inflammatory and immune-suppressive. Examples include that alcohol reduces the development of destructive rheumatoid arthritis (RA), an autoimmune disease characterized by severe joint inflammation (48, 49). Moderate alcohol intake was also inversely associated with the risk of systemic lupus erythematosus (SLE), another autoimmune disease (50, 51). Alcohol attenuation of the chronic inflammatory diseases resembles the therapeutic effect of steroids. However, the molecular link between alcohol immunosuppression and steroid immunosuppression has not been clearly defined. To our best knowledge, this report provides the first evidence suggesting that alcohol employs an alternative mechanism to activate GR signaling, which may contribute to the long-observed steroid-like effect of alcohol on cell immune response.

The non-ligand-bound GR, induced by alcohol, interacts with GREs and activates *gilz*, suggesting that the “naked” form of GR has biological activities. This finding presents an exception to the current paradigm that GR has to couple with ligands in order to have transacting capacity. Alcohol is a versatile solvent, miscible with water and with organic solvents. This amphiphilic property grants this molecule the ability to perturb protein–protein interactions that are reliant on weak bonds, such as in the GR complex. GR protein without any GC coupling binds GREs specifically (Figure 4), indicating that ligand binding is not necessarily required for GR to interact with GREs. This finding predicts the possibility that any reagents or condition that affects the stability of the GR complex might trigger GR signaling without GC. As a matter of fact, elevations of pH and temperature, shear stress, and other circumstances are reported to induce GR nuclear translocation in a ligand-independent manner (21, 52, 53).

GILZ acts as an endogenous carrier that transmits GC therapeutic actions to achieve anti-inflammation and immunosuppression (31, 54–56). GILZ expression is reduced or even absent in various inflammatory disorders, such as chronic rhinosinusitis, Crohn’s disease, and atherosclerosis (55, 57, 58). These lines of evidence indicate that suppression of GILZ expression predisposes the host to hyperinflammatory diseases. Contrarily, transgenic expression of GILZ in mice protects the animals from colitis (59). Exogenous supplement of a GILZ peptide suppresses autoimmune encephalomyelitis (60). In the current study, we demonstrated that depletion of GILZ in MM6 cells abolished the alcohol suppressive effect on inflammatory cytokine production elicited by LPS challenge. These data suggest that the GR-GILZ pathway is critical to alcohol anti-inflammation and immunosuppression.

It was noticed that the RT-qPCR data (Figure 1) demonstrate that Dex stimulated GILZ expression by ~27-fold, while the luciferase assay data (Figure 2) show that Dex only enhanced luciferase expression by ~2.3-fold in the case of GILZ long promoter (pGL-Long-GILZp). Such a disparity could result from the following inequalities: (1) unlike the natural GILZ promoter, the isolated GILZ promoter used to drive luciferase expression is a minimal promoter that lacks all the enhancers; (2) RT-qPCR is a more sensitive detection method than enzymatic assay; and (3) RT-qPCR measures mRNA abundance, while luciferase assay measures luciferase activity. Nevertheless, the two results are consistent even though not directly comparable.

In Figure 3, the ADH inhibitor fomepizole was used to inhibit alcohol metabolism. We found that with fomepizole application, significantly more GR nuclear translocation was observed, suggesting that alcohol facilitates such a process. We are aware that there exist multiple alcohol oxidation pathways. The most important pathway is ADH-mediated, as ADHs constitute the dominant enzyme system involved in ~80% of alcohol oxidation (61). The second pathway is through the cytochrome P450 system, also called microsomal alcohol oxidizing system or “MEOS” (mostly CYP2E1), which oxidizes only a small fraction of alcohol in the case of acute alcohol exposure. Future experiments are warranted to define how blocking MEOS affects GR translocation.

As illustrated in the proposed model (Figure 7) GR signaling can be activated *via* the canonical ligand binding or the non-canonical alcohol induction. However, the number of GREs required for each activation appears to be different. We found GRE1 and 2 are sufficient to support a full GC activation of *gilz* (Figure 2). Nevertheless, all five GREs are needed for a full alcohol activation of *gilz*. It is noteworthy that *in vivo* alcohol exposure may lead to concurrent activations of both pathways. It is unknown how the two GR-activation pathways mutually influence each other, which may collectively define the ultimate outcome of the HPA-GR signal transduction under the condition of alcohol exposure.

In summary, we have demonstrated that alcohol activates GR signaling and *gilz* expression through a non-canonical GR–GRE interaction. This GR-activation mechanism contributes to alcohol modulation of cell inflammatory and immune responses.

## Author Contributions

HN conducted the experiments and analyzed the data. JW made lentiviral constructs and performed the RT-qPCR analyses. SJ did GR gene-knockout and established the GR−/− MM6 cell line. PM and SN provided advice to the project. GW conceived the idea, designed the experiments, and wrote the paper.

## Conflict of Interest Statement

The authors declare that the research was conducted in the absence of any commercial or financial relationships that could be construed as a potential conflict of interest.

## Footnotes

^1^http://www.multicrispr.net/.

^2^http://crispr.mit.edu/.
