# Supplementary material for: Non-canonical Glucocorticoid Receptor Transactivation of gilz by Alcohol Suppresses Cell Inflammatory Response
Source: Front Immunol. 2017 Jun 7;8:661. doi: 10.3389/fimmu.2017.00661 (PMC5461336; doi:10.3389/fimmu.2017.00661)
Supplement: Supplementary file 1 [file Presentation_1.PDF]

SUPPLEMENTAL DATA

Figure S1

```
-1940   GTGCAGAGGGCAAATTAATATTAATATATCAGGGAATTCTGATACCAGTTAAGCTCCTGATTTAAGAGGT
           Forward Primer
-1870   TTTTTTTTTTAGTGAATGTTCTTGATGACCCATAAGTATAGCCTGCACTTTGTTCTGTCTACTACACATG
                   GRE-5                                   GRE-4
-1800   TCTTAGTGCAAACACCGTGTTCAGAGAGGTTGTGTCTTAAAGTTAACAGAATGTCCTGAGATCATTATTG
                                                       GRE-3
-1730   TCTCCCACCTAAAGTGAGATGAAGAAAAACACCATCCCGGGCTAGTGCAGCAGGTTTACTTAGTTCTATT
        GGTCAGTACTTTGCAATATAATGGCTCTTGTACACATATTATCACCACTGGAATCCACCAATAGAGCACC
-1590   ACACCATAGCCAAAAATGTTGGCTGTTCAGTGGCATAACTGCCCTGGAAGCCGGGAGGAAATCAAGGCCT
                                                                Eta
-1520   TCAGACTGCATTTGGCTGGGGCCTTGGCCAGGGTGTTAGTGCCAGGCACCTGGTTAAGATAAATGTCCTG
                LSF                 NF-1                          GATA
-1450   AAGCAAACAATAATTACCTTCTTTGCTGATAAGAGGCCCCAGTACTTTTCCAATAGCATGATTGCGTTAG
                                    GATA              NFAT                CRE
-1380   GATCTTTCTTTGGACCACAGCCTGTGGAGCACTGATTCATGGGTACTGGCCTTAACTTCATCCAAACTGT
                     NF-1
-1310   TGTATATGTGGTGGAACCCAATGTTCTCCTTTGGTCCTGAGTTGGTGTAAATAATATTCCCGGTTGCATC
                         GRE-2                                               CRE
-1240   ACCTGTCCCTGCCCCTCTTGCTCCTTCTGGTGGAAATTCTGCTTTTGTCCACCAGGTGGCCTCACTTAGG
        AAGGAGCAGAAGAAAAGGACCTCAAGTCTCTGGTGCCTTTATTTTTTTTCCCTCAGCTTGAGGCTGAATA
                                                      NFAT
-1100   TAAATAAGATATATTTTAATGGAAACGTGTAACTTTTATTATGGAACAATCACCTCATCTTGTTTATGGT
                                                                    FHRE3
-1030   TTGGAGTCTGTTTTTCCTGAAGTACCTCTAATGCTGTGCATGGCTGGGCCCATCACAGGGCAGGTGTTTT
        CCTGGGATCGTTTATATAAATGTATACCTTTGGTTTGGAGTGTTATTGTCATTCGCTGTTTTAAAGAAAA
-890    GTAACAAAGTTGTGTTTTTAAAAAATAGAGACACGGGATCCTGGATAATGTTGAATGTATTTTCTTCAAA
        GAGTAGAATGTGGAGACTAAAGAGAAAAGGGCTGGCATCTTGTGGTCACTCCAGTGCTCACTCCAATACC
-750    CTGTCTCCTCCTCACCTCCTCTGTCACCCACCTAGCTGCTCTCCCATACATGTTCACCCACTCACCTCAG
        CTCCCGGACACCCTCTCCTCCATACATATGCATCTGTAGATTAGCATGGACCTGTGCATGCATTACATGC
-610    ATGTCACAGATCTGTACATGTGCCGGTCCACAGACAAGTACTCCCACACCTGAACATTCAGAGGAAGGGG
                                                                STAT6   Eta
-540    CAAGTGGATGAGTGCTGGGGTTCAGGGCAGGTCGGCAGGCCAGCTGGCTGGTGAGGGGAAAGCTGAGTTG
        ATTAGAGAGTGAATGTGCAGGTGACAAAGTATGTGTGCAGGGGAGAGACTGAGAGGGCAGTGACTGGGAA
-400    GAGGGTGAGGCCGTGATGGTGAGGGCGCGTGTCCAGGGAGTATGACATGGGAGAGCATGCAAAGCCATGG
                                  C-Myc
-330    GGGCATGCTGGCTGCCACCTGAAGAAGAGCTTTTCAAACTAGCGCTGTTCTTTACTGAATGCCCTCTGCC
                   LSF                           GRE-1
-260    TCTTGTTAGGGCATTTGTATTTCTTATTTCTCTAGAAATCAGCTCCAGTTTGGTTTTTATCGATCTCCAG
                     Oct-1           STAT-6
-190    AGCCTTCTTTGGAGATGCCAGTTGGTACAAGAAAGTGCTGAACTGTTTACAGTCCCAGCCTAAGGTTCAG
              SRF                           FHRE-1
-120    GGAGGGGATGTGGTTTAACTGGGCCACAAAGCCCGGTACAGGACTCATTTGCATGGCCCCTGACGCCATG
                                                                     CRE
-50     TGACGCAGCCGGCTCCTCCTATATAAAGAAGCAGGAGCCAAAATATCTCC
            CRE                TATA
+1      GAGTCTGGGTTGGACTGGCG
        Reverse Primer
```

Fig. S1. Primary sequence of the cloned GILZ promoter and its potential cis-acting elements. The primary sequence of the cloned GILZ promoter is displayed and the nucleotides are numbered relative to the transcriptional start site, as described in the main context. The identified *cis*-elements are highlighted in colors. GRE: Glucocorticoid responsive element; CRE: cAMP response element; FHRE-1: Forkhead responsive element; SRE: Serum response element; STAT-6: Signaling transducer and activator of transcription 6; Oct-1: Octamer 1; NFAT: Nuclear factor of activated T cell; NF-1: Nuclear factor 1; GATA: GATA Box; LSF: Late simian virus 40 transcription factor; Ets: ETS domain binding site. Forward and Reverse primers were the primers used to amply the promoter region.
