# Supplementary material for: Non-canonical Glucocorticoid Receptor Transactivation of gilz by Alcohol Suppresses Cell Inflammatory Response
Source: Front Immunol. 2017 Jun 7;8:661. doi: 10.3389/fimmu.2017.00661 (PMC5461336; doi:10.3389/fimmu.2017.00661)
Supplement: Supplementary file 2 [file Presentation_2.PDF]

SUPPLEMENTARY DATA

Figure S2

```
Mut-GR  AGAACTGTATTTTGATATTCCTTTGACAGTTAAATCATAACACTGTTCTTCCCCTTCTTTAGCCCCAGCATGAGACCAGATGTAAGCTCTCCTCCATC
 WT-GR  AGAACTGTATTTTGATATTCCTTTGACAGTTAAATCATAACACTGTTCTTCCCCTTCTTTAGCCCCAGCATGAGACCAGATGTAAGCTCTCCTCCATC

        CAGCTCCTCAACAGCAACAACAGGACCACCTCCCAAACTCTGCCTGGTGTGCTCTGATGAAGCTTCAGGATGTCATTATGGAGTCTTAACCGGAGCAA
        CAGCTCCTCAACAGCAACAACAGGACCACCTCCCAAACTCTGCCTGGTGTGCTCTGATGAAGCTTCAGGATGTCATTATGGAGTCTTAAC--------

        CGGTCAGATGGCAGGCATTAATCCACACTGTGGGGATGAATCACCTCCCCAAGACGGCATCCACTTACAACAACCCACTTCTCATCTTGTGGAAGCTG
        --------------------------------------------------------------------------------------TTGTGGAAGCTG

        TAAAGTTTTCTTCAAAAGAGCAGTGGAAGGTAGTGTGTGTTTTGAAGAGTTTATTTTTCCTCTACTTGGTTTTCATTTCTCAGGGTGGATTTTGAAAT
        TAAAGTTTTCTTCAAAAGAGCAGTGGAAGGTAGTGTGTGTTTTGAAGAGTTTATTTTTCCTCTACTTGGTTTTCATTTCTCAGGGTGGATTTTGAAAT

        TTCCATTATATGCAAAGCCCATGAAAGGCT
        TTCCATTATATGCAAAGCCCATGAAAGGCT
```

Fig. S2. Sequence comparison between GR-/- (Mut-GR) and GR+/+ (WT-GR) MM6 cell clones. The top line displays the GR-Exon-3 and its adjacent sequences for Mut-GR (GR-/-) clone, and WT-GR (GR+/+) clone. The GR Exon-3 sequence is underlined. The sgRNA target sequence is highlighted in green color and the PAM site in purple color. The Mut-GR sequence has a 94bp insertion at the Cas9 DNA double strand break. BLASTing Mut-GR sequence against the human genome gives an otherwise perfect match with WT GR Exon 3 sequence on chromosome 2 (GRCh38.p7 Primary Assembly, 230612240 to 230612333).
