# Supplementary material for: Non-canonical Glucocorticoid Receptor Transactivation of gilz by Alcohol Suppresses Cell Inflammatory Response
Source: Front Immunol. 2017 Jun 7;8:661. doi: 10.3389/fimmu.2017.00661 (PMC5461336; doi:10.3389/fimmu.2017.00661)
Supplement: Supplementary file 3 [file Presentation_3.PDF]

SUPPLEMENTARY DATA

Figure S3

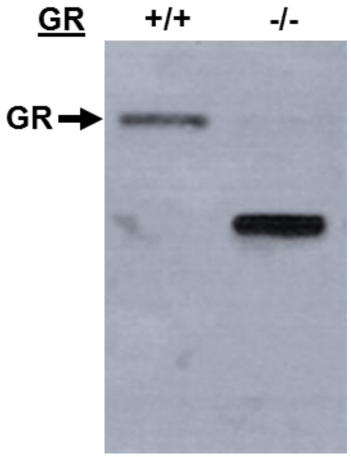

Fig. S3. Western blot analysis of GR+/+ and GR-/- clones. The WT-GR (GR+/+) and the Mut-GR (GR-/-) MM6 cell clones were subjected to Western blot assay using a GR polyclonal antibody (Santa Cruz Biotechnology, Inc.). The Mut-GR clone had a premature ending of GR translation and produced a truncated GR protein, as seen in a significantly small size.
