# Supplementary material for: Non-canonical Glucocorticoid Receptor Transactivation of gilz by Alcohol Suppresses Cell Inflammatory Response
Source: Front Immunol. 2017 Jun 7;8:661. doi: 10.3389/fimmu.2017.00661 (PMC5461336; doi:10.3389/fimmu.2017.00661)
Supplement: Supplementary file 4 [file Presentation_4.PDF]

SUPPLEMENTARY DATA

Figure S4

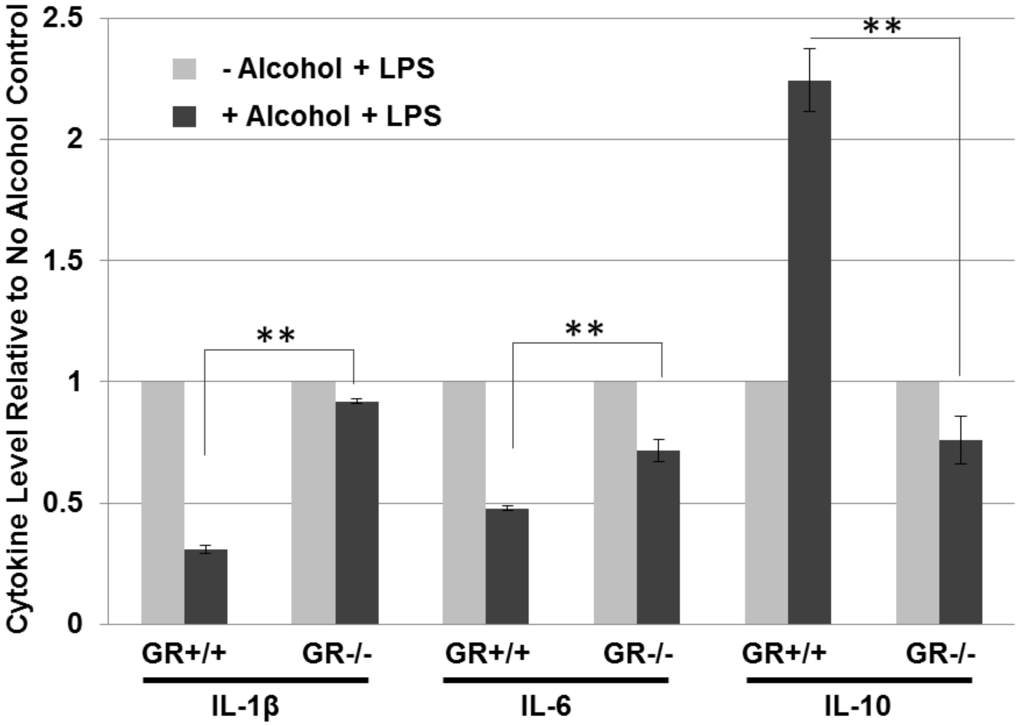

Fig. S4. LPS-stimulated cytokine production by GR+/+ and GR-/- MM6 cells with or without alcohol exposure. The GR+/+ and GR-/- MM6 cell clones were exposed to 0 mM or 50 mM alcohol, and stimulated with LPS (1 µg/ml) for 24 hours. The culture media were collected for inflammatory cytokine measurements by ELISA. As shown, alcohol exposure substantially suppressed pro-inflammatory cytokine (IL-1β and IL-6) production and promoted anti-inflammatory cytokine production in GR+/+ cells. However, such alcohol effects were diminished in GR-/- cells. These data suggest that GR is crucial in alcohol modulation of cell inflammatory response. Student's t-test was used to judge any statistical significance between the comparing groups (** $p<0.01$, $n=3$).
